# The antitumor activity of human Vγ9Vδ2 T cells is impaired by TGF-β through significant phenotype, transcriptomic and metabolic changes

**DOI:** 10.3389/fimmu.2022.1066336

**Published:** 2023-01-19

**Authors:** Chirine Rafia, Clément Loizeau, Ophélie Renoult, Christelle Harly, Claire Pecqueur, Noémie Joalland, Emmanuel Scotet

**Affiliations:** ^1^ Nantes Université, Inserm UMR 1307, CNRS UMR 6075, Université d’Angers, CRCI2NA, Nantes, France; ^2^ LabEx IGO “Immunotherapy, Graft, Oncology”, Nantes, France

**Keywords:** human, Vγ9Vδ2 T lymphocytes, cancer, regulation, TGF-β

## Abstract

Despite significant advances, the eradication of cancer remains a clinical challenge which justifies the urgent exploration of additional therapeutic strategies such as immunotherapies. Human peripheral Vγ9Vδ2 T cells represent an attractive candidate subset for designing safe, feasible and effective adoptive T cell transfer-based therapies. However, following their infiltration within tumors, γδ T cells are exposed to various regulating constituents and signals from the tumor microenvironment (TME), which severely alter their antitumor functions. Here, we show that TGF-β, whose elevated production in some solid tumors is linked to a poor prognosis, interferes with the antigenic activation of human Vγ9Vδ2 T cells *in vitro*. This regulatory cytokine strongly impairs their cytolytic activity, which is accompanied by the induction of particular phenotypic, transcriptomic and metabolic changes. Collectively, these observations provide information for better understanding and targeting the impact of TME components to regulate the antitumor activity of human T cell effectors.

## Introduction

In the wake of revolutionary cancer immunotherapies, adoptive transfer strategies harnessing effector functions of particular immune cell subsets have been proposed. Among the most attractive candidates, human peripheral Vgamma9Vdelta2 (Vγ9Vδ2) T lymphocytes, which represent 1 to 10% of blood CD3^+^ T lymphocytes in healthy adults, contribute to host defenses against various infections and malignancies (1, 2). These conserved and non-alloreactive unconventional T lymphocytes are selectively activated in a TCR/CD3-dependent, but MHC-independent, manner by non-peptidic small metabolites of isoprenoid synthesis pathways called PAg (phosphoantigen), such as IPP (isopentenyl pyrophosphate). This peculiar and exquisitely specific stress sensing process, which remains ill defined, also involves BTN (butyrophilin)3A/BTN2A molecules which are expressed at the surface of target cells (3–5). The sensitization of target cells with elevated pinocytic activity by pharmacological NBP (aminobisphosphonate) compounds, such as zoledronate, which inhibit a key IPP-degrading enzyme of the mammalian mevalonate pathway, upregulate the reactivity of Vγ9Vδ2 T lymphocytes (6). This *Self*-targeted reactivity process needs to be tightly controlled by a set of molecules, such as NK (natural killer) receptors, adhesion molecules, Toll-*like* receptors or immune checkpoint inhibitors. The activating NKG2D (natural killer group 2, member D) receptor is expressed by cytotoxic immune cell subsets, including Vγ9Vδ2 T lymphocytes. Once engaged by stress-induced determinants that are barely, if not, expressed by healthy cells, these receptors play a key role in sustaining TCR/CD3-induced reactivities and subsequent antitumor functions, such as perforin/granzyme-mediated cytolysis. Vγ9Vδ2 T cells integrate activation and inhibition signals to deliver, or not, rapid and strong functional responses, such as proliferation, cytolysis and cytokines (ie. TNF (tumor necrosis factor)-alpha, IFN (interferon)-gamma) release (7).

Importantly, Vγ9Vδ2 T lymphocytes can sense and eliminate a broad range of human circulating and solid tumor cells. The range of tumor cell lines detected by Vγ9Vδ2 T cells, which was initially restricted to circulating tumors (eg. hematopoietic tumors), has been next extended to various solid tumors (e.g. renal cancer and colon carcinomas) (8). The development of clinical grade synthetic agonist molecules such as NBP, PAg or anti-BTN agonist antibodies enabled the implementation of passive or active Vγ9Vδ2 T cell immunotherapies with modest therapeutic effects (9). However, while several groups initially reported tumor infiltration by human γδ T cells, additional work evidenced that some tumor-associated γδ T cells display pro-tumor functions (10, 11). This result highlights the role played by the TME (tumor microenvironment) on tumor-infiltrated γδ T cells in fostering immune escape. Among TME factors, TGF-β (transforming growth factor-beta), a highly conserved multi-functional cytokine, is secreted by different cell types (eg. lymphocytes, macrophages) and regulates various biological functions of tumor and T cells (12, 13). Indeed, different studies have shown its strong suppressive activity within solid tumors by inducing lymphocyte cell death or converting effector T cells into T helper 17 or T regulatory lymphocytes (see (12) for a review).

On the basis of the strong and well described impact of TGF-β on antitumor T cells reactivities, we investigated its influence on the human effector Vγ9Vδ2 T cell subset. Our results evidenced that antitumor functions of Vγ9Vδ2 T cells are greatly impaired by TGF-β. More importantly, they demonstrate that this impairment is not only profound, as it affects the transcriptome and the metabolism of Vγ9Vδ2 T lymphocytes, but also long lasting.

## Materials & methods

### Reagents, human Vγ9Vδ2 T and tumor cells

Human Vγ9Vδ2 T lymphocytes were amplified from PBL (peripheral blood lymphocytes) obtained from healthy donor blood samples provided by the Etablissement Français du Sang (EFS, Nantes, France) and after Ficoll density centrifugation (Eurobio). For specific *ex vivo* expansions of peripheral allogeneic human Vγ9Vδ2 T lymphocytes, PBL were incubated with 3 µM of BrHPP (bromohydrin pyrophosphate, kindly provided by Innate Pharma (Marseille, France)) in RPMI medium supplemented with 10% heat-inactivated fetal calf serum, 2 mM L-glutamine (Gibco), 100 μg/mL streptomycin (Gibco), 100 IU/mL penicillin (Gibco), and 100 IU/mL human recombinant IL-2 (Proleukin, Novartis). After 4 days, cell cultures were supplemented with 300 IU/mL IL-2. On day 21, the purity of cultures was checked by flow cytometry and the population with a purity >90% were conserved. Human Raji (Burkitt’s lymphoma) and SKOV3 (ovarian carcinoma) were cultured in complete RMPI (Roswell Park Memorial Institute, Gibco) and DMEM (Dulbecco’s Modified Eagle’s Medium, Gibco) medium, supplemented with 10% heat-inactivated fetal calf serum (Gibco), 2 mM L-glutamine, 100 μg/mL streptomycin, 100 IU/mL penicillin, respectively. Human primary GBM (glioblastoma multiforme) cultures were obtained after mechanical dissociation of surgical resection tumor samples from patients. All procedures involving human patients were performed in accordance with the ethical standards of the ethic national research committee and with the 1964 Helsinki declaration and its later amendments or comparable ethical standards. Informed consent was obtained from all individual patients included in this study. Primary cultures were established and stored at −180°C. Cell-frozen vials were grown in defined medium (DMEM/Ham F12 (Gibco), 2 mM L-glutamine, N2- and B27-supplement (Gibco), 2 μg/mL heparin (Sigma Aldrich), 20 ng/mL EGF and 25 ng/mL bFGF (Peprotech), and 100 IU/mL penicillin and 100 μg/mL streptomycin at 37°C in a humidified atmosphere with 5% CO2. All experiments were performed with GBM cells in culture for less than 3 months and cells were routinely checked for mycoplasma contamination. Recombinant human transforming growth factor β1 (premium grade, Miltenyi Biotec) was diluted and used at the indicated concentrations.

### Flow cytometry

TGF-β-treated or -untreated human Vγ9Vδ2 T lymphocytes were stained with FITC-labeled anti-human TCR Vδ2 mAb (#IMMU389, Beckman Coulter) and APC-labeled anti-human CD3ϵ mAb (#UCHT1, Beckman Coulter), to check the purity of the populations. T lymphocytes were phenotyped using PE-labeled anti-NKG2A (#Z199, Beckman Coulter), PE-labeled anti-NKG2D (#ON72, Beckman Coulter), PE-labeled anti-CD85j/ILT2 (#HP-F1, Beckman Coulter), APC-labeled anti-ICAM1 (#HA58, Biolegend). Acquisition was performed using an Accuri C6 PLUS flow cytometer (BD Biosciences) and the collected events were analyzed using FlowJo software (Treestar). Examples of raw cytometry data and gating strategies are shown in **Supplemental Figure S1**.

### Functional assays *in vitro*

For CD107a surface mobilization assays, primary human GBM cells or cell lines were sensitized, or not, with zoledronate (Zometa, Sigma) overnight at indicated concentrations before incubation with human Vγ9Vδ2 T lymphocytes (effector to target ratio 1:1). Cocultures were performed in RPMI medium containing 5 µM monensin (Sigma) and Alexa Fluor 647-labeled anti-human CD107a mAb (#H4A3, Biolegend). After 4 h, human Vγ9Vδ2 T lymphocytes were collected and stained with a FITC-labeled anti-human TCR Vδ2 mAb (#IMMU389, Beckman Coulter) and analyzed by flow cytometry. For perforin/granzyme stainings, fixed Vγ9Vδ2 T cells (PBS-PFA 4%), were incubated with permeabilization buffer and stained with fluorochrome-labeled anti-human perforin and granzyme B mAbs (#dG9, eBioscience; #GB11, BD Biosciences). The acquisition was performed using a FACSCalibur flow cytometer (BD Biosciences) and the events were analyzed using the FlowJo software (Treestar, Ashland, OR). To perform cytotoxic assays, tumor cells were previously treated with zoledronate overnight at indicated concentrations before loading with ^51^Cr (75 µCi/10^6^ cells) for 1 h. After washes, loaded tumor cells were cocultured with TGF-β-treated or -untreated human Vγ9Vδ2 T lymphocytes (effector to target ratio 10:1) for 4 h. The activity of ^51^Cr released in supernatants was measured using a MicroBeta counter (Perkin Elmer). Specific target cell lysis was calculated as follows: % Lysis=[(Experimental release-Spontaneous release)/(Maximum release-Spontaneous release)] x 100. Spontaneous and maximum release values were obtained by adding culture medium or 1% Triton X−100, respectively, to ^51^Cr-loaded target cells in the absence of human Vγ9Vδ2 T lymphocytes. To measure Vγ9Vδ2 T cells viability, lymphocytes were harvested after 1, 3 or 5 days in culture, in the presence or not of TGF-β. Cells were next washed and stained with 7-AAD (BD Bioscience). Cytometry acquisition were performed using an Accuri C6 PLUS.

### Transcriptional analyses

Briefly, RNA was prepared from dry pellets of resting Vγ9Vδ2 T lymphocytes (10^6^ cells/>95% purity) which have been collected following treatment (3 days), or not, with TGF-β. RNA was extracted and purified using the NucleoSpin RNA^+^ isolation kit from Macherey-Nagel. Both the quality and the amount of extracted RNA were checked using Nanodrop OneC. DGE-sequencing was performed by the BIRD core facility (SFR Bonamy, Nantes University); data were analyzed using R programming tools. All sequencing data and protocol used are available under GEO (deposit number #GSE213696).

### Metabolic analyses

Mitochondrial oxygen consumption (OCR) and extracellular acidification (ECAR) rates were measured in non-buffered medium containing cystine (0.2 mM) supplemented with glucose (5 mM), pyruvate (1 mM), and glutamine (2 mM) using an XFp (8 wells) Analyzer (SeaHorse Bioscience). Briefly, TGF-β-treated and -untreated resting human Vγ9Vδ2 T lymphocytes were washed in the analysis medium and plated at 8.10^5^ cells per well using Cell-Tak (Corning) at 22.4 µg/mL. Plates were next placed at 37°C to equilibrate for 1 h prior to starting the analysis. Measurements were first taken every 10 min. After 3 cycles, BrHPP was injected and 3 measures were taken rapidly, every 3 min, followed by 5 cycles of 10 min. PMA+ionomycin were injected and the same protocol of measurement was applied than after BrHPP. Specific mitochondrial respiration in the presence of a stimuli (BrHPP, PMA-ionomycin) was calculated as follows: difference between the mean of the 3 values of OCR in the absence of substrate and the mean of the 4 values of OCR after injection of the substrate. Analyses of the obtained values were performed using the Wave 2.6 software (Agilent).

### Statistical analyses

Data are expressed as mean ± SD and were analyzed using GraphPad Prism 6.0 software (GraphPad Software). Two-way ANOVA and Mann & Whitney tests were calculated to assess or not for significant differences.

## Results

### The levels of TGF-β and γδ TCR transcripts are both linked to the prognosis of human glioblastoma (GBM) cancer patients

**Figure 1:**
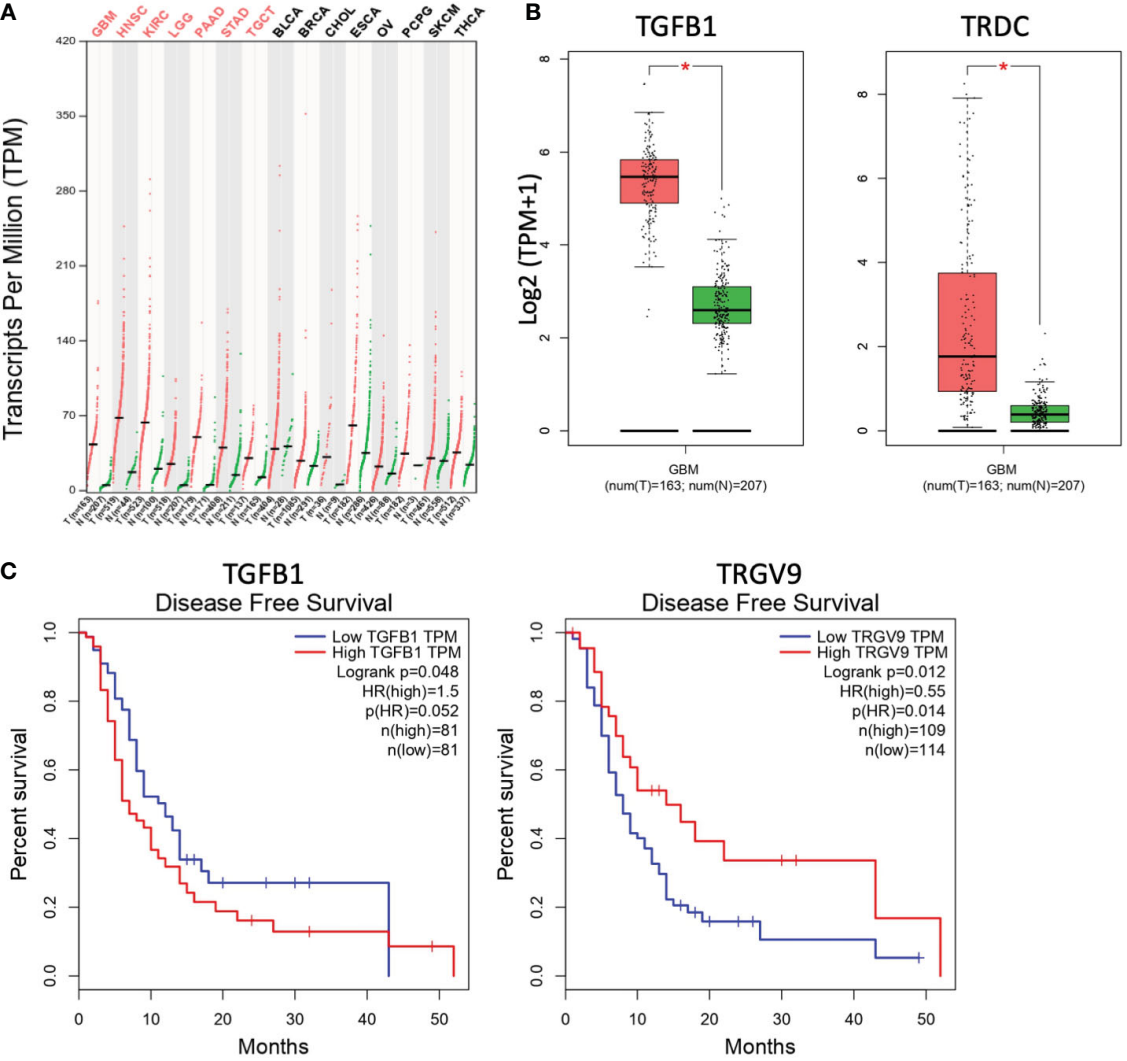
Levels of TGF-β and γδ TCR transcripts are differently linked to the prognosis of glioblastoma tumors. **(A)** The abundance of human TGF-β transcripts (TPM, *transcripts per million*) across various indicated cancers (*T, red*) and the normal tissues associated (*N, green*) was analyzed and compared by matching TCGA and GTEx data. GBM, glioblastoma multiforme; HNSC, head and neck squamous cell carcinoma; KIRC, kidney renal clear cell carcinoma; LGG, brain lower grade glioma; PAAD, pancreatic adenocarcinoma; STAD, stomach adenocarcinoma; TGCT, testicular germ cell tumors; BLCA, bladder urothelial carcinoma; BRCA, breast invasive carcinoma; CHOL, cholangiocarcinoma; ESCA, esophageal carcinoma; OV, ovarian serous cystadenocarcinoma; PCPG, pheochromocytoma and paraganglioma; SKCM, skin cutaneous melanoma; THCA, thyroid carcinoma. *Red abbreviations*, tumors with significantly increased TGF-β transcripts, as compared to normal tissues. *n*, number of clinical samples. **(B)** Comparative analyses of transcript levels [Log2 (TPM+1)] of TGF-β1 (TGFB1, *left*) and TCR δ chain (TRDC, *right*) in GBM tumors (*red*) *vs* normal tissues associated (*green*). *p<0.05. **(C)** Compared disease-free survival curves for GBM patients with high (*red*) *vs* low (*blue*) levels of transcripts of TGF-β1 (*left*; TGFB1; cutoff-high 50%) or TCR Vγ9 chain (*right;* TRGV9; cutoff-high 25%). Data from GEPIA (http://gepia.cancer-pku.cn/).

The abundance of TGF-β transcripts was analyzed *in silico* within several human tumors using GEPIA (*Gene Expression Profiling Interactive Analysis*, http://gepia.cancer-pku.cn/) which is an interactive web-based tool developed to deliver fast and customizable functionalities based on TCGA (*The Cancer Genome Atlas*) and GTEx (*Genotype-Tissue Expression*) data (14). These transcriptomic analyses evidenced elevated quantities of TGF-β transcripts in some human tumors (*red*), such as glioblastoma (GBM), lower grade glioma (LGG) or head and neck squamous cell carcinoma (HNSC), as compared to associated normal tissues (*green*) (**Figure 1A**). Interestingly, these analyses did not only evidence increased levels of TGF-β1 transcripts (TGFB1) in GBM, they also showed the elevated presence of Vδ TCR chain transcripts (TRDC) in these brain tumors (**Figure 1B**). Increased expressions of *TGFB2* and *TGFB3* genes were also detected (*data not shown*). Moreover, high levels of TGF-β1 transcripts (TGFB1) in GBM tumors are correlated to a reduced disease-free survival of GBM patients. These survival values are also associated to low levels of Vγ9 TCR chain (TRGV9) transcripts (**Figure 1C**). Taken together, these results indicate that elevated levels of TGF-β transcripts are found in different human cancers, including GBM tumors. These brain tumors also contain γδ TCR transcripts, whose reduced levels are associated to dismal prognosis values.

### TGF-β inhibits the antitumor cytotoxic reactivity of human Vγ9Vδ2 T cells

**Figure 2:**
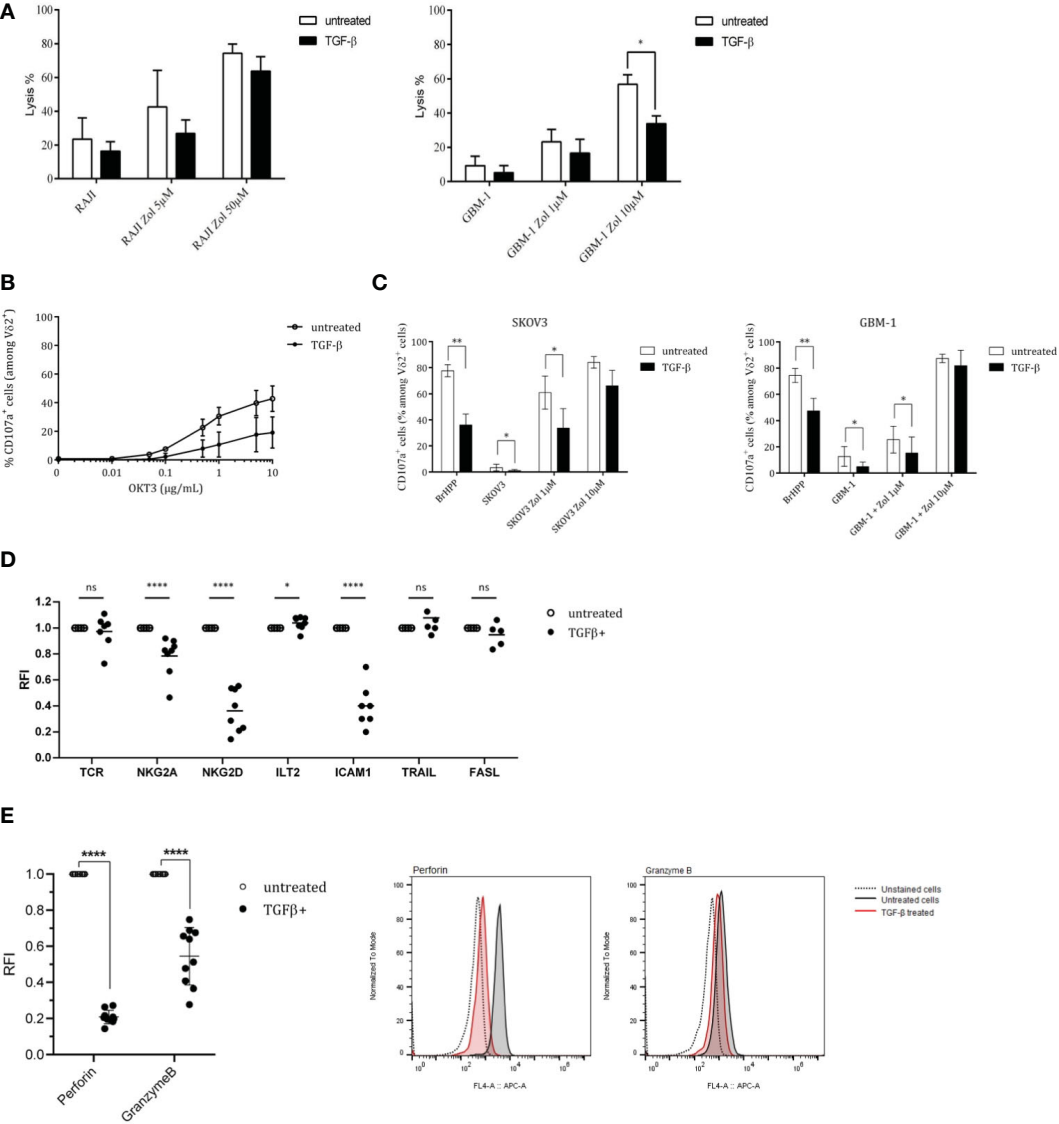
TGF-β inhibits both antitumor cytotoxicity and antigenic activation of human Vγ9Vδ2 T cells. PBL-derived Vγ9Vδ2 T cells were pretreated, or not, with TGF-β (10 ng/mL) for 72 h. **(A)** Cytotoxic reactivities of TGF-β-treated (*closed symbols*) *vs* untreated (*open symbols*) human Vγ9Vδ2 T lymphocytes against human tumor cells (Raji, *left*; GBM-1, *right*) using ^51^Cr-release assays. Target cells have been sensitized, or not, with zoledronate (Zol) at 5 μM and 50 µM. Results are expressed as % of cytotoxicity (mean ± SD). **(B)** and **(C)** Frequency of CD107a^+^ cells among TGF-β-treated (*closed symbols*) *vs* untreated (*open symbols*) TCR Vδ2^+^ lymphocytes (%) after stimulation with increasing concentrations of anti-CD3 mAb (OKT3) **(B)** or zoledronate (Zol)-treated, or not, human SKOV3 (*left*) or GBM-1 (*right*) tumor cells **(C)**. E/T ratio 1/1. Positive control: BrHPP (3 µM). n≥5 for **(B)** and **(C)**, mean ± SD. **(D)** and **(E)** Expression levels of activation-related molecules (TCR, NKG2A, NKG2D, ICAM1, TRAIL, FASL) **(D)** and cytolytic (Perforin, Granzyme B) molecules **(E)** by TGF-β-treated (*closed symbols*) *vs* untreated (*open symbols*) TCR Vδ2^+^ lymphocytes measured by flow cytometry. n=8 for **(D)** and **(E)**. RFI, Relative Fluorescence Intensity. Mann-Whitney *p<0.05; **p<0.01; ****p<0.001. ns, not significant.

Considering both elevated levels of TGF-β transcripts in brain human tumors with a dismal prognosis and the potential therapeutic role of Vγ9Vδ2 T cells against GBM cells (15, 16), we investigated the inhibitory effect induced by TGF-β. While TGF-β induced a slight, but not significant, decrease of the cytolytic activity (^51^Cr-release assays) of Vγ9Vδ2 T cells against RAJI tumor cells (**Figure 2A**, left), this cytokine reduced such a lytic activity to greater extent against GBM-1 cells (**Figure 2A**, right). CD107a surface expression assays were next performed in either non-specific activation (α-CD3 mAb, **Figure 2B**) or tumor cells co-culture (SKOV3/GBM-1; **Figure 2C**) conditions. The results show that CD107a expression levels are reduced upon TGF-β treatment, whatever the activation strength and condition. Such a reduced cytolytic activity against tumors was also observed following short-time (24 h) exposures (*data not shown*). Interestingly, strong antigenic activation conditions (zoledronate, 10 µM) reduced the intensity of this inhibition.

In an effort to investigate such diminished activation potential and antitumor reactivities, the levels of expression of key selected surface molecules were analyzed and compared by flow cytometry with a focus on markers playing a role in the antitumor reactivity such as TCR, ICAM-1/CD54, activatory (NKG2D) or inhibitory (NKG2A/CD94; ILT2) NK receptors as well as cytolytic pathways associated molecules (perforin (perf), granzyme B (GmzB), Fas Ligand (FASL), TNF-related apoptosis-inducing ligand (TRAIL) (**Figure 2D**). The most striking effect of this TGF-β exposure was the strongly reduced expression of the activating receptor NKG2D, the cell adhesion molecule ICAM-1 and perforin/granzyme B which represent key contributors of antitumor cytolytic pathways used by Vγ9Vδ2 T cells. TGF-β exposure did not significantly affect the expression of TCR, FASL and TRAIL molecules and slightly affected the expression of inhibitory receptors. Following dose-responses analyses to determine optimal TGF-β concentrations for inducing a significant reduction of NKG2D expression (**Supplemental Figure S2A**), the kinetics of effects induced by TGF-β was established, at the protein level, by measuring the expression of NKG2D at different timepoints following addition of TGF-β (**Supplemental Figure S2B**). The expression of NKG2D remained stable for about 4 h in the presence of TGF-β and started to decrease after 8 h, by reaching its lowest expression levels between 24 h and 72 h. Interestingly, our next experiments showed that, following exposure to TGF-β for different durations and washes, the impact of this cytokine on NKG2D expression was lasting for days, with a partial restoration of initial expression levels 120 h after withdrawing TGF-β (**Supplemental Figure S2C**).

The intracellular levels of expression of cytolytic molecules from TGF-β-treated and untreated Vγ9Vδ2 T cells were measured and compared (**Figure 2E**). These analyses focused on perforin/granzyme B, FAS-L and TRAIL pathways which constitute the major pathway contributing to the antitumor cytolytic activity of Vγ9Vδ2 T cells. TGF-β-treated Vγ9Vδ2 T cells expressed significantly lower levels of perforin and granzyme B, as compared to untreated conditions (RFI, Relative Fluorescence Intensity=20% and 50%, respectively). Taken together, these results show that TGF-β significantly inhibits the antigenic reactivity as well as the antitumor cytolytic potential and activity of human Vγ9Vδ2 T cells.

### The transcriptome of human Vγ9Vδ2 T cells is significantly modified upon TGF-β exposure

**Figure 3:**
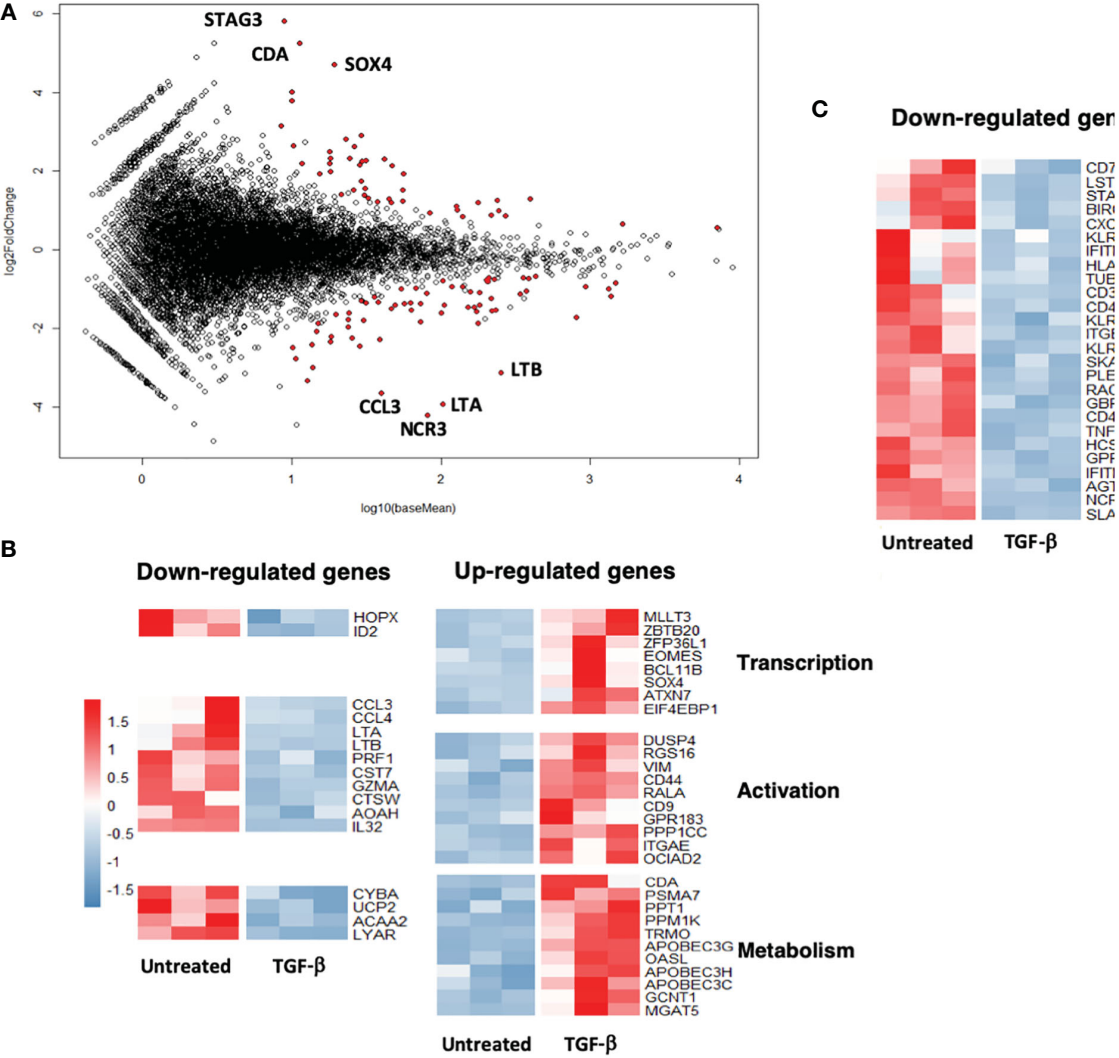
TGF-β exposure impacts the transcriptome of human Vγ9Vδ2 T cells. Human PBL-expanded Vγ9Vδ2 T cells were pretreated, or not, with TGF-β (10 ng/mL) for 72 h prior to RNA extraction. Comparative DGEseq analyses were performed on bulk RNAseq samples, data were analyzed using the R software. MA plot **(A)** and heat maps **(B)** and **(C)** representing genes (indicated functions) that have been retrieved as differentially expressed following TGF-β exposure or not (*Untreated*). Colored scale, fold change expression. *Down-regulated genes* (*blue*); *Up-regulated genes* (*red*).

To check whether inhibition of the antitumor Vγ9Vδ2 T cells reactivity induced by TGF-β could also be linked to transcriptome modifications, comparative DGE-seq analyses from bulk RNA-seq data have been performed. RNA samples were prepared from PBL-derived Vγ9Vδ2 T cells exposed, or not, for 3 days to TGF-β *in vitro*. 89 genes (40 upregulated, 49 downregulated) were found differentially expressed (fold change > 2, adjusted p value < 0.05) between TGF-β-treated and -untreated groups (**Figure 3A**). Interestingly, MA plot analysis, which represents log fold-change versus mean expression of genes between TGF-β-treated and -untreated Vγ9Vδ2 T cells, shows that well-expressed down-regulated genes mainly encode for surface-bound receptors such as NCR3/NKP30/CD337 or inflammatory factors such as CCL3/MIP-1-alpha, LT (lymphotoxin) A/TNF-α, LTB/TNF-C. Conversely, these analyses did not evidence a strong up-regulation of well-expressed genes following TGF-β exposure. Most of the differentially expressed genes were related to either effector function, transcription, activation or metabolic functions (**Figure 3B**) (upregulation, red; downregulation, blue) upon TGF-β exposure. This cytokine diminished the expression levels of important genes related to effector functions such as CD40LG or CXCR6 described to regulate tissue trafficking of T lymphocytes (**Figure 3C**). These analyses show that the TGF-β induces the establishment of a particular transcriptional program in exposed Vγ9Vδ2 T cells which could be distinguished from regulatory or exhausted T cell signatures (17).

### TGF-β alters the metabolic functions of Vγ9Vδ2 T cells

**Figure 4:**
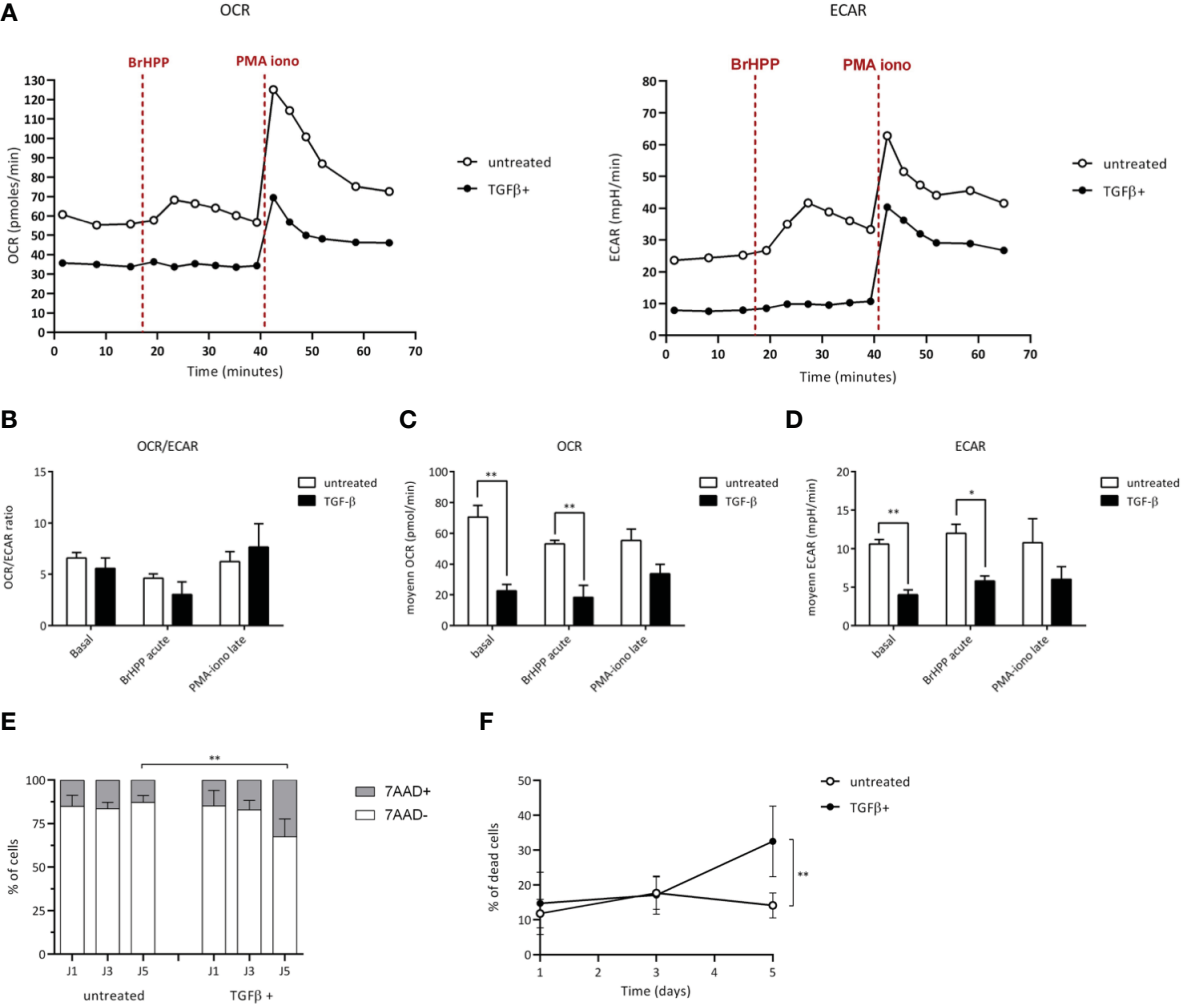
TGF-β alters the metabolism of Vγ9Vδ2 T cells. Vγ9Vδ2 T cells were treated (*closed symbols*), or not (*open symbols*), with TGF-β (10 ng/mL) for 72 h. Metabolic activities of T lymphocytes were measured using Seahorse XFp. **(A-D)** Monitoring of mitochondrial oxygen consumption rate (OCR) and extracellular acidification rate (ECAR) were performed at resting state, after BrHPP stimulation or PMA-ionomycin injection as described in the *Materials and Methods* section. **(A)** Representative experiments. Rates were calculated as follows: difference between the mean of the 3 values in the absence of substrate and the mean of the 4 values after injection of the substrate, as well as a ratio of the 2 for each condition **(B-D)**. **(E)** and **(F)** Cell death induced by TGF-β added within the media of Vγ9Vδ2 T cells (10 ng/mL for 72 h) was measured by measuring fluorescent non-viable cells following 7-AAD staining after the indicated culture timepoints days of culture. n=6, mean ± SD. Mann-Whitney *p<0.05; **p<0.01. PMA-iono: PMA+ionomycin (*positive control*).

A growing number of studies shows that TME can alter the functions of infiltrating T cell effectors by affecting their metabolic activity (18, 19). To further investigate the existence of similar effects induced upon TGF-β exposure of human Vγ9Vδ2 T cells which was suggested by our transcriptome analyses, the energy metabolism was measured and compared in different conditions through real-time live cell analysis using Seahorse Extracellular Flux analyzer that enables direct quantification of mitochondrial respiration and glycolysis. T cells were monitored at a resting state and after specific antigenic (BrHPP) or non-specific (PMA+ionomycin) activations (**Figure 4A**). In resting conditions, Vγ9Vδ2 T cells exposed to TGF-β (*closed symbols*) showed a weaker oxygen consumption (OCR, oxygen consumption rate) and media acidification rate (ECAR, extracellular acidification rate), as compared to their untreated counterparts. As expected, both specific and non-specific activations increased ECAR and OCR levels of control Vγ9Vδ2 T cells (*untreated*). However, a strong specific antigenic activation (BrHPP, 3 µM) of TGF-β-exposed Vγ9Vδ2 T cells induced barely detectable, if any, increase of ECAR or OCR metabolic activities. Moreover, upon exposure to TGF-β, OCR and ECAR levels were significantly lower in Vγ9Vδ2 T cells, as compared to untreated T lymphocytes. Non-specific activation increased these metabolic levels without reducing these differences between these exposure conditions (**Figures 4B-D**). Moreover, TGF-β exposure reduces the viability of Vγ9Vδ2 T cells, as compared to untreated conditions (**Figures 4E, F**). Taken together, the results show that TGF-β, which is produced in various TME of solid tumors, strongly impairs the metabolism of human Vγ9Vδ2 T cells by both reducing their mitochondrial respiration, their glycolytic activity and their viability which are key parameters for antitumor functions (20).

## Discussion

Our results show that TGF-β, which transcription is detected at high levels in brain tumors with a poor prognosis, interferes with the antigenic activation of human Vγ9Vδ2 T cells *in vitro*. Importantly, they evidence that this tumor-produced regulatory cytokine strongly impairs their antitumor cytolytic reactivity, through multiple phenotypic, transcriptomic changes as well as metabolic alterations.

Regarding tumor surveillance, the immune system orchestrates with broad molecular and cellular networks for sensing and eliminating tumor cells. Numerous studies focused on innate-*like* T cell subsets including γδ T cells, and particularly on non-alloreactive peripheral human Vγ9Vδ2 T cells which represent promising effector candidates for designing novel immunotherapies. The implication of Vγ9Vδ2 T cells in antitumor immune response is supported by the identification of tumor-infiltrated γδ T cells, which might be considered as a prognosis tool for some oncological indications (21, 22). However, the clinical efficacy of Vγ9Vδ2 T cell-based therapies is still disappointing. Although their conserved and MHC-independent specificity is expected to overcome issues related to antigen presentation and recognition, their efficacy against solid tumors remains limited and hampered by various immunological, biochemical and tumor-*linked* constraints. Once T cells have successfully reached and infiltrated the tumors, they need to evolve within a strong immunoregulatory environment that aims at both taming their antitumor functions and promoting tumor development.

TME is a complex network constituted by cancer-associated fibroblasts, myofibroblasts, extracellular matrix, immune/inflammatory cells, blood, and vascular networks as well as signaling components including cytokines, chemokines and growth factors. Previous studies have shown the sensitivity of Vγ9Vδ2 T cells to two TME-produced immunosuppressive factors: PGE (prostaglandin E) 2 and TGF-β (23, 24). PGE2 potently suppresses the proliferation, cytokine production, and cytotoxic capacity of gamma delta T cells. TGF-β, a secreted dimeric multifunctional cytokine that signals *via* specific receptors on plasma membranes, contributes to cancer progression by its ability to promote epithelial-to-mesenchymal transition of cancer cells, angiogenesis, and immune evasion (25). We showed that TGF-β exposure severely impacts the cytolytic activity of human Vγ9Vδ2 T cells. Interestingly, this inhibition was durable *in vitro* (up to 72 h) and reversible in the absence of the cytokine. TGF-β exposure reduces the expression of lytic molecules (eg. perforin, granzyme B), as well as surface molecules mediating this antitumor reactivity such as NKG2D, as described for NK and CD8^+^ T cells (12). NKG2D was extensively described as a key receptor for sustaining the reactivity of various lymphoid subsets, including human Vγ9Vδ2 T cells, against several tumor cells. Supporting this observation, we previously evidenced the major role played by NKG2D for the elimination of human mesenchymal GBM tumor cells by allogeneic human Vγ9Vδ2 T cells (16) which can infiltrate GBM (26). Therefore, in such pathological contexts, one could expect that NKG2D-controled antitumor Vγ9Vδ2 T cell reactivities would be altered by TGF-β which transcription is elevated in brain tumors (27, 28) as previously described for NK and CD8^+^ T cells (29).

Studies reported opposite effects of this cytokine in which the antitumor activity of TGF-β-conditioned Vγ9Vδ2 T cells is potentiated and could be used for improving their efficacy in adoptive transfer therapies (30, 31). This highlights the complex pleiotropic effects of some regulatory cytokines on effector T cells, according to the context of exposure (eg. sensitization of resting T cells *versus* conditioning for amplification) which might introduce several different parameters such as the cellular environment, the kinetics as well as the differentiation status/plasticity of T cells. As shown here, TGF-β does not only directly inhibit the antitumor reactivity of infiltrated Vγ9Vδ2 T cells but also induces specific modifications of their transcriptional program and phenotype. Comparative RNA-seq analysis performed on resting Vγ9Vδ2 T cells exposed to TGF-β, did not show any polarization towards Th17 (eg. STAT3, IL-22), Th9 (eg. IRF4, IL-9) or regulatory (eg. FoxP3) functional profiles. The lack of TGF-β-induced regulatory functions was also confirmed in T cell proliferation assays *in vitro* (*data not shown*). TGF-β exposure affects the expression of genes either implicated in lymphocyte activation, transcription effector processes (downregulation). Interestingly, as already observed for NK cells (32), the expression of NKG2D as well as NCR3/NKp30 is strongly impacted by TGF-β exposure. We showed that this cytokine impacts particular metabolic pathways in human Vγ9Vδ2 T cells. Both glycolysis and mitochondrial respiration, which are key metabolic anti-tumor effector pathways (33) are strongly abated upon cytokine exposure. This plunges TGF-β-exposed Vγ9Vδ2 T cells in a hypoxia-*like*-induced state through raising their activation threshold, thus leading to a dramatic reduced reactivity against tumor cells.

Altogether, the results indicate that TGF-β, a cytokine that is massively produced in the TME of various solid tumors, acts as a potent taming cytokine on resting PBL-expanded human Vγ9Vδ2 T cells, at different levels. It does not only directly target the key NKG2D axis, to inhibit the antitumor cytolytic activity but also affects their transcriptional and metabolic program to reduce their viability and functional activity. This should be taken in account for designing improved Vγ9Vδ2-based cancer immunotherapies, by proposing genome editing technologies (eg. CRISPR-Cas9) for preparing engineered Vγ9Vδ2 T cells that can effectively counter tumor escape promoted by TME-derived factors (34). The inhibition of TGF-β signaling pathway could be proposed for improving Vγ9Vδ2 T cell-based therapies by using either inhibitors, blocking antibodies or T cells expressing a dominant negative TGFBR2 receptor (see (13) for a review). Another attractive strategy would rely on the use of inhibitors of TGF-β signaling molecules in cancer cells, such as ubiquitin-specific peptidase 8 (USP8) which is a metastasis enhancer and an active deubiquitinase in aggressive tumors (eg. breast) (35). USP8 inhibition has been proposed to alleviate T cell (such as Vγ9Vδ2) exhaustion and thus to improve the efficacy of immunotherapies.

## Data availability statement

The datasets presented in this study are deposited in the GEO repository, accession number: GSE213696.

## Author contributions

CR, NJ, CP and ES conceived and designed the experiments. CR, CL, OR performed the experiments. CR, CL, OR, CH and CP analyzed the data. CR and ES wrote the manuscript. CR, OR, CH, CP, NJ and ES reviewed the manuscript. All authors contributed to the article and approved the submitted version.

## Supplementary material

Supplementary Figure 1 Gating strategies selected for the analysis by flow cytometry of the impact of TGF-β on human Vγ9Vδ2 T cells. *Left*, selection of Vδ2^+^ T cells (Vd2 subset, purity indicated=95.4%) within PBMC. *Right up*, CD107a expression on activated Vδ2^+^ T cells; *Right, low*, NKG2D expression levels on TGF-β-treated (*purple histogram*) or untreated (*blue histogram*) Vδ2^+^ T cells. Negative control, *gray histogram*. Percentage values are indicated in the quadrants.

Supplementary Figure 2
**(A)** Normalized expression of NKG2D measured by flow cytometry at the surface of human Vγ9Vδ2 T cells incubated for 3 days with indicated increasing doses of recombinant human TGF-β. **(B)** Kinetics of expression of NKG2D expressed at the surface of human Vγ9Vδ2 T cells at different time-points, following addition of TGF-β (10 ng/mL). Cell surface stainings were measured by flow cytometry. **(C)** NKG2D expression at the surface of human Vγ9Vδ2 T cells incubated with recombinant human TGF-β (10 ng/mL) at the indicated time-points (ctrl, negative control). NKG2D expression levels were next measured and normalized, at the indicated time-points after washing the cells. n>5 Mann-Whitney tests *p<0.05; **p<0.01.

## Glossary

**Table:**

bFGF	basic fibroblast growth factor
BTN	butyrophilin
BrHPP	bromohydrin pyrophosphate
CCL3	C-C motif ligand 3
CD	cluster of differentiation
CD40LG	CD40 ligand
CRISPR	clustered regularly interspaced short palindromic repeats
CXCR6	C-X-C motif chemokine receptor 6
DGE	differential gene expression
DMEM	Dulbecco’s Modified Eagle’s Medium
ECAR	extracellular acidification
EGF	epidermal growth factor
FASL	Fas ligand
FITC	fluorescein isothiocyanate
GBM	glioblastoma multiforme
GEPIA	Gene Expression Profiling Interactive Analysis;
GmzB	granzyme B
GTEx	genotype-tissue expression
ICAM	inter cellular adhesion molecule
IFN	interferon
IL-2	interleukin-2
ILT2	Ig-like transcript 2
IPP	isopentenyl pyrophosphate
IRF4	interferon regulatory factor 4
LGG	low-grade glioma
LT	lymphotoxin
mAb	monoclonal antibody
MHC	major histocompatibility complex
MIP-1	macrophage inflammatory protein-1
NCR3	natural cytotoxicity
