# Supplementary figures and images for: The antitumor activity of human Vγ9Vδ2 T cells is impaired by TGF-β through significant phenotype, transcriptomic and metabolic changes
Source: Front Immunol. 2023 Jan 19;13:1066336. doi: 10.3389/fimmu.2022.1066336 (PMC9893774; doi:10.3389/fimmu.2022.1066336)

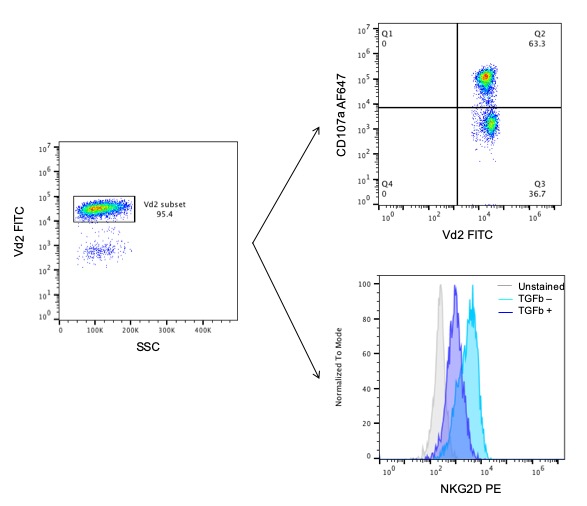

Supplement: Supplementary Figure 1 — Gating strategies selected for the analysis by flow cytometry of the impact of TGF-β on human Vγ9Vδ2 T cells. Left, selection of Vδ2+ T cells (Vd2 subset, purity indicated=95.4%) within PBMC. Right up, CD107a expression on activated Vδ2+ T cells; Right, low, NKG2D expression levels on TGF-β-treated (purple histogram) or untreated (blue histogram) Vδ2+ T cells. Negative control, gray histogram. Percentage values are indicated in the quadrants. [file Image_1.jpeg]

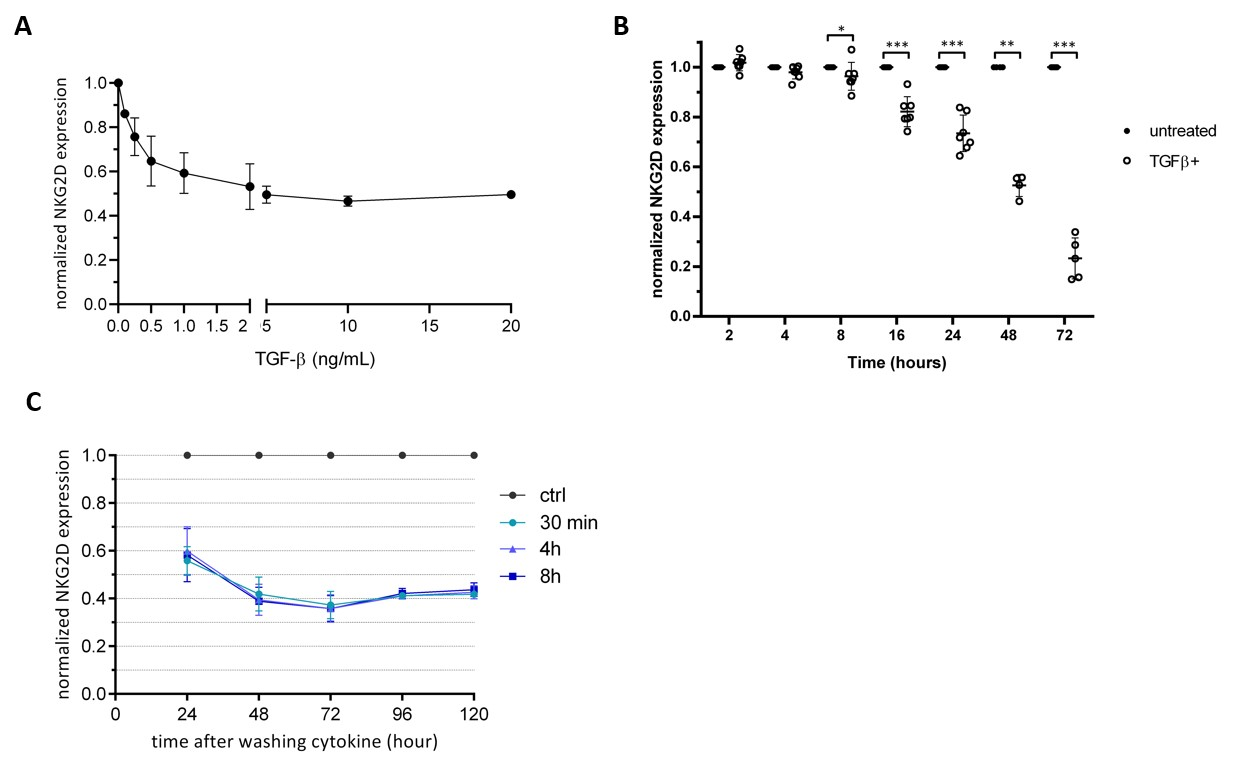

Supplement: Supplementary Figure 2 — (A) Normalized expression of NKG2D measured by flow cytometry at the surface of human Vγ9Vδ2 T cells incubated for 3 days with indicated increasing doses of recombinant human TGF-β. (B) Kinetics of expression of NKG2D expressed at the surface of human Vγ9Vδ2 T cells at different time-points, following addition of TGF-β (10 ng/mL). Cell surface stainings were measured by flow cytometry. (C) NKG2D expression at the surface of human Vγ9Vδ2 T cells incubated with recombinant human TGF-β (10 ng/mL) at the indicated time-points (ctrl, negative control). NKG2D expression levels were next measured and normalized, at the indicated time-points after washing the cells. n>5 Mann-Whitney tests *p<0.05; **p<0.01. [file Image_2.tiff]
